# Evaluating Clinical Outcomes and Physician Adoption of Telemedicine for Chronic Disease Management: Population-Based Retrospective Cohort Study

**DOI:** 10.2196/66499

**Published:** 2025-04-28

**Authors:** Ido Peles, Lena Novack, Shosh Peleg, Eran Levanon, Michal Gordon, Mariya Abayev, Victor Novack, Shlomi Codish

**Affiliations:** 1 Clinical Research Center Soroka University Medical Center Ben-Gurion University of the Negev Beer-Sheva Israel; 2 Faculty of Health Sciences Ben-Gurion University of the Negev Beer-Sheva Israel; 3 Soroka University Medical Center Beer-Sheva Israel

**Keywords:** telemedicine, chronic disease management, COVID-19, health care utilization, virtual care, emergency department referral, hospitalization, natural experiment, clinical effectiveness, chronic disease, management, effectiveness, outpatient, clinical outcome, emergency department, cohort, physician

## Abstract

**Background:**

In recent years, the use and impact of telemedicine for providing health care services to patients has increased, reducing the requirement for physical, in-person encounters.

**Objective:**

This study aimed to compare the use of telemedicine for outpatient visits versus in-person visits across different medical specialties; assess its association with clinical outcomes; and examine the influence of patient and physician characteristics on telemedicine use in a large, tertiary, teaching hospital.

**Methods:**

The study cohort consisted of adult patients who attended outpatient clinics in five medical fields (psychiatry, endocrinology, nephrology, hemato-oncology, and gastroenterology) in 2019 and survived until the beginning of 2020. Telemedicine use during the period of 2019-2021 was the main exposure of interest. The primary outcomes were emergency department (ED) referrals and hospitalizations. The analysis used multivariate mixed models and subgroup analysis by patient demographic characteristics, chronic disease medical fields, and physicians’ characteristics.

**Results:**

The cohort included 32,445 patients. In 2019, a total of 99.6% (82,668/83,000) of visits were in person, and by 2020-2021, a total of 22.6% (10,850/48,120) of patients had used telemedicine. Telemedicine patients were slightly older (standardized mean difference=0.281; *P*<.001), with a higher comorbidity burden than in-person patients or patients without visits (standardized mean difference=0.328; *P*<.001). Presurge telemedicine users had higher rates of ED referrals (incidence rate ratio [IRR] 1.15, 95% CI 1.09-1.21) and hospitalizations (IRR 1.14, 95% CI 1.08-1.20) than in-person visit users. These ratios remained stable during the surge (IRR 1.1, 95% CI 1.06-1.16 and IRR 1.12, 95% CI 1.05-1.19, respectively), with no evidence of worsening outcomes for telemedicine users relative to in-person care. Health care providers with higher telemedicine use had reduced rates of ED referrals (IRR 0.85, 95% CI 0.79-0.91) and hospitalizations (IRR 0.78, 95% CI 0.72-0.84) than providers with lower telemedicine use.

**Conclusions:**

This study provides insights into telemedicine use patterns and their association with clinical outcomes in chronic disease management. Our findings suggest that the increase in telemedicine use was not associated with a rise in ED referrals or hospitalizations when compared to in-person visits. It highlights the importance of health care providers’ perspectives and use of remote visits. Telemedicine should be tailored to individual patient-physician needs, considering the nature of the patient’s disease.

## Introduction

Telemedicine refers to the use of technology to provide health care services remotely without the need for in-person encounters [1]. Although telemedicine has demonstrated benefits in diverse clinical settings and patient populations, its implementation did not significantly accelerate until the COVID-19 pandemic, leading to widespread adoption worldwide [2-4].

Besides the pandemic, several factors that emerged in the last decade have also contributed to the growth in the use of telemedicine. These factors include advances in information and communication technology, widespread high-speed internet access including the use of mobile devices, and the growing adoption of electronic health records [5,6].

Numerous studies have been performed to understand the use, efficacy, and best practices for the implementation of telemedicine care across several disease and specialty areas [3]. Outpatient consultation or remote ambulatory care is one of the fields of greatest use [4]. Chronic disease management via telemedicine has become significant in various fields such as cardiology, endocrinology, rheumatology, nephrology, and other areas where longitudinal metrics of patient data are essential for evaluating trends in quality measures (eg, hemoglobin A_1c_ [HbA_1c_] and serum creatinine) [2]. These findings align with other recent research that found telemedicine management to be at least as effective as in-person care in such fields [6-8].

The evolution of telemedicine over the years has been remarkable. In the United States, telemedicine adoption in hospitals rose from 46% in 2017 to 72% in 2021, driven primarily by larger teaching hospitals leading the transformation [9]. Similarly, in China, a regional telemedicine platform experienced substantial growth in remote consultations, providing critical benefits to underserved regions and older adults [10]. These trends reflect a broader recognition of telemedicine’s potential to revolutionize health care delivery. For instance, the American College of Cardiology and other medical societies have emphasized that telemedicine not only improves access to care but also enhances patient satisfaction, reduces manpower requirements, and fosters equity in health care delivery [11,12].

Nevertheless, alongside the potential benefits of telemedicine, there are challenges, for instance, the absence of the physical examination, which is an inherent part of the visit and diagnosis. In addition, challenges related to administrative, reimbursement, legal, privacy, security, and regulatory domains arise from the fact that technological innovation often outpaces advances in policy [13].

Recently, the Israeli Ministry of Health announced the promotion and implementation of telemedicine as one of its goals [14]. Understanding the effectiveness, strengths, and shortcomings of telemedicine for various chronic diseases and patient populations can inform decision makers of health care policy on how best to implement and maximize its benefits. To comprehensively evaluate the impact of telemedicine, we assessed use patterns among physicians and their association with patient outcomes. Telemedicine in this study was implemented through telephone consultations integrated into routine outpatient care, focusing on follow-ups and chronic disease management, with its application verified through hospital records. This approach allowed us to comprehensively evaluate telemedicine’s integration into care delivery and its implications for patient outcomes and health care delivery systems.

In this research, we aimed to compare the use of telemedicine versus in-person visits in different fields of medicine, as well as patients’ and physicians’ characteristics as they relate to the use of telemedicine in the outpatient clinics of a large, tertiary, teaching hospital.

## Methods

### Overview

The Israeli National Health Insurance Law provides universal health care coverage, requiring all citizens to join 1 of 4 nonprofit health insurance organizations. Clalit Health Services, the largest Israeli health insurance organization, insures a little over half of the population and provides care across different geographic regions; access to health services is similar for residents in each region [15]. In the southern region of Israel, with a population of over 750,000 residents (8.2% of the Israeli population), Clalit Health Services insures approximately 67% of the residents. Soroka University Medical Center (SUMC), a 1191-bed teaching hospital, is the sole provider of tertiary care in the region. This setup, with a single hospital serving a large region, allowed for population-based analysis, with minimal patient loss to follow-up and reduced referral bias.

This population-based, retrospective, observational, cohort study included adults (aged ≥18 years)

who were treated at the outpatient clinics of SUMC in five medical fields (psychiatry, endocrinology, nephrology, hemato-oncology, and gastroenterology) in 2019 and survived until the beginning of 2020.

The exposure of interest was telemedicine use during the pandemic period of 2019-2020. We divided the study population into two groups: patients who used telemedicine at least once and those who did not. The primary outcomes of the analysis were emergency department (ED) referrals and hospitalizations. The secondary outcomes were measurements used for monitoring chronic diseases including thyroid-stimulating hormone (TSH), triiodothyronine, thyroxine (T4), HbA_1c_, creatinine, urine microalbumin-creatinine ratio, and blood pressure measurements. We hypothesized that telemedicine use would not be associated with inferior clinical outcomes for individual patients. The sample size of 32,445 participants was determined to provide sufficient power to detect a minimal effect size of 1.2% in the primary outcomes (ED referrals and hospitalizations), with 80% statistical power and a significance level of .05. These target numbers were determined based on the assumptions extracted from previous studies [7,8].

For descriptive statistics, we initially assessed telemedicine utilization rates across various clinics before and during the COVID-19 pandemic. Using descriptive statistics, we gained insights into the extent of telemedicine adoption in each clinic. Data were summarized as means and SDs for normally distributed quantitative variables, medians and ranges for nonnormally distributed quantitative variables, and percentages for qualitative variables.

Next, we performed bivariate analyses to identify differences between patients and physicians who opted for telemedicine and those who did not. We also explored bivariate associations between demographic and clinical variables and primary outcomes, including ED referrals and hospitalizations. For categorical variables, we used a chi-square test, with the Fisher exact test when needed. For continuous variables, distributions were assessed using the Shapiro-Wilk test and Q-Q plots; then, we used a 2-tailed *t* test for normally distributed variables and a Mann-Whitney *U* test for nonnormally distributed variables.

**Figure 1:**
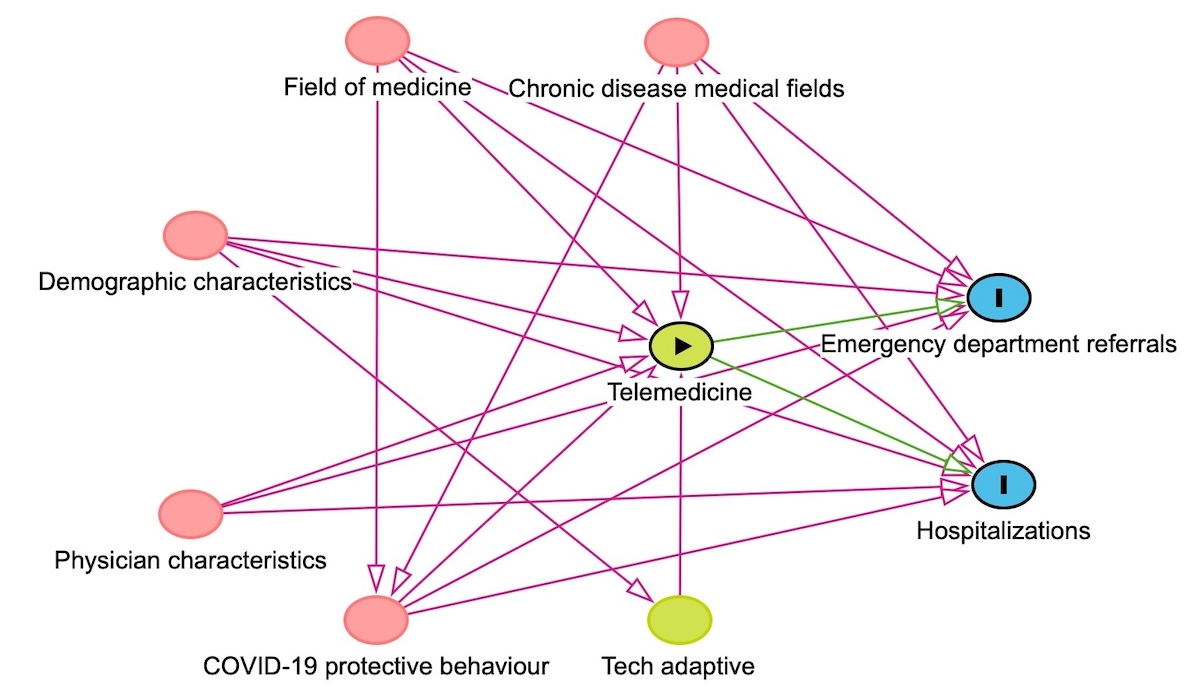
Directed acyclic graph of the assumptions on the relationship between variables.

Based on the results of the bivariate analysis, we applied multivariable mixed models. We presented point estimates of association using the incidence rate ratio (IRR) or relative risk (RR) with their corresponding 95% CI. IRR was derived using Poisson regression models with an offset for the follow-up period, accounting for the rate of ED referrals and hospitalizations per patient per unit time. RR was computed by comparing the probability of the outcome occurring in telemedicine patients relative to in-person patients within the same time frame, controlling for confounding variables. These models were adjusted for covariates selected based on the bivariate analysis results and clinical or epidemiological significance. A directed acyclic graph (DAG) was used to represent the associations between variables. The DAG connecting telemedicine use with ED referrals and hospitalizations is depicted in Figure 1. In the DAG, arrows represent associations between variables, while unconnected variables indicate no direct association. All statistical analyses were performed with consideration of the DAG framework, including chosen covariates to minimize the bias of the estimands of telemedicine use on study outcomes. The baseline covariates included in the models were demographic characteristics (age, sex, ethnicity, socioeconomic score, and Charlson Comorbidity Index [CCI]), physician characteristics (age, sex, career status, and distance from hospital), medical field, chronic disease monitoring measurements, and COVID-19–related behaviors (annual primary physician visits, COVID-19 vaccinations, and infections). Data on physicians' technology orientation were unavailable and therefore not included in the modeling. However, we believe it is not associated with the outcomes.

We conducted subgroup analyses by stratifying patients based on chronic diseases and demographic variables. This approach allowed us to investigate potential variations in outcomes within specific patient groups. Finally, we scrutinized disparities in telemedicine use among different physicians. We characterized their use patterns and categorized them into quartiles based on the percentage increase in telemedicine usage during the surge. Subsequently, we compared the results across the various groups of physicians.

RStudio (version 1.4.1717; R Foundation for Statistical Computing) was used for these analyses. A 2-sided *P* value <.05 was considered statistically significant for all analyses.

### Ethical Considerations

The study was approved by the SUMC ethics committee (reference 0368-21). All clinical investigations were conducted according to the principles expressed in the Declaration of Helsinki. The ethics committee approval exempted the study from informed consent due to the retrospective data collection that maintained subject confidentiality. Informed consent was waived by the institutional review board at SUMC. Patient records were anonymized and deidentified prior to analysis.

## Results

**Figure 2:**
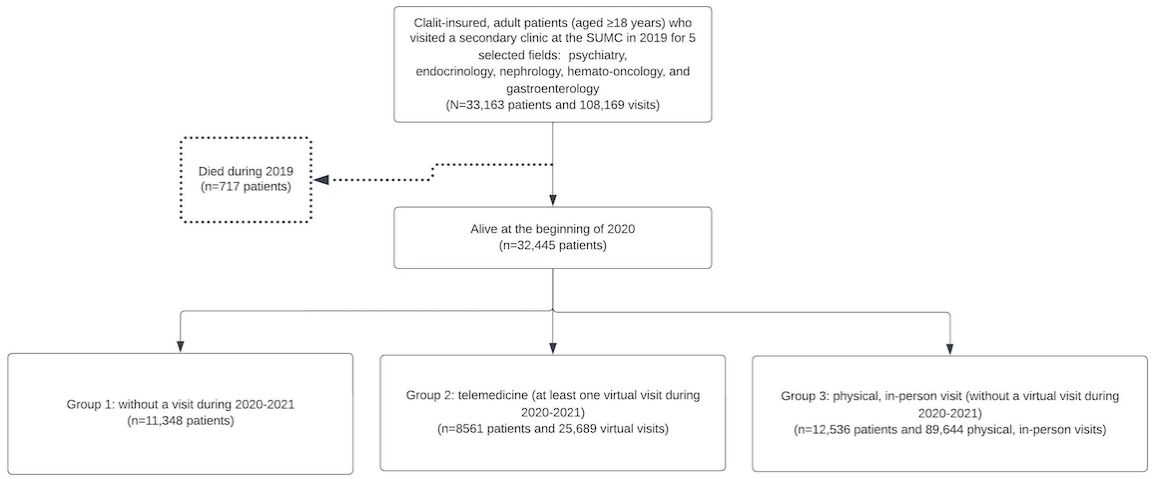
Study flow diagram. SUMC: Soroka University Medical Center.

We identified 108,169 visits to the hospital’s outpatient clinics by 33,162 patients in 2019 (Figure 2). At the beginning of 2020, a total of 32,445 patients were alive, and we classified them into three groups—those with no visits during 2020-2021 (11,348 patients), those with at least one remote consultation during that period (8561 patients), and those with only in-person visits (12,536 patients). In 2019, almost all care (82,668/83,000, 99.6%) occurred in person, while during 2020-2021, a total of 22.6% (10,850/48,120) of visits were via telemedicine. This notable increase across all medical specialties, with the highest use seen in nephrology and diabetes mellitus clinics at 36.7% (1354/3689) and 32.6% (1194/3662), respectively. Telemedicine use in the remaining specialties were as follows: 26.9% (1029/3825) in endocrinology, 22.4% (3170/14,176) in hematology, 13.7% (906/6618) in psychiatry, 21% (3046/14,544) in oncology, and 9.4% (151/1606) in gastroenterology.

Demographic and clinical characteristics of patients receiving physical in-person care; patients receiving telemedicine services; and those in the control group, which had no visits during 2020-2021, are presented in Table 1. Patients in the in-person and telemedicine groups were older on average (mean ages of 60.8, SD 17.4 years and 62, SD 17.6 years, respectively) compared with the control group (mean 52.9, SD 20 years; standardized mean difference [SMD]=0.281). Sex distribution was similar across the groups, with female patients comprising 60% (7527/12,536) of the physical, in-person group; 57.2% (4901/8561) of the telemedicine group; and 60.1% (6754/11,348) of the control group (SMD=0.041). Most patients in all groups were Jewish, with a slightly higher percentage in the telemedicine group (7465/8561, 87.2%) compared with the in-person (10,577/12,536, 84.4%) and control group (9225/11,348, 82.1%). The telemedicine group had a higher proportion of patients who immigrated before 1991 (3293/8561, 38.5%) compared with the in-person (4300/12,536, 34.3%) and control groups (3034/11,348, 27%; SMD=0.195). The mean age-adjusted CCI also differed across the groups (4.66 for remote consultations, 4.33 for in-person visits, and 3.21 for no visits; SMD=0.328). Smoking prevalence was similar across all groups; it was slightly higher in the telemedicine group (2962/8561, 34.6%) than in the physical, in-person group (3884/12,536, 31%).

COVID-19 infection rates were similar across the groups. However, the median number of COVID-19 vaccinations was slightly higher in the physical, in-person group (3, IQR 0-3) and telemedicine group (3, IQR 2-3) than the control group (2, IQR 0-3; SMD 0.209). The annual number of primary care physician visits before and during 2020-2021 were similar between the in-person group and telemedicine group, with a median of 15 (IQR 10-21) and 16 (IQR 11-23) visits in 2019, respectively, and 16 (IQR 11-23) and 14 (9.5-20) visits in 2020-2021 (SMD=0.233 and 0.379, respectively).

**Table 1:** Patient characteristics.

Characteristics		Physical, in-person patients (N=12,536)	Telemedicine patients (n=8,561)	Control group (n=11,348)	SMD^a^	
**Age (years)**						0.281
	Mean (SD)	60.8 (17.4)	62.0 (17.6)	52.9 (20)		
	Median (IQR)	64.0 (49-74)	66.0 (50-75)	54.0 (35-69)		
	<35, n (%)	1620 (12.9)	1073 (12.5)	2847 (25.3)		
	35-50, n (%)	1933 (15.4)	1285 (15)	2236 (19.9)		
	50-75, n (%)	6840 (54.6)	4514 (52.7)	4516 (40.2)		
	>75, n (%)	2143 (17.1)	1689 (19.7)	1642 (14.6)		
**Sex, n (%)**						0.041
	Female	7527 (60)	4901 (57.2)	6754 (60.1)		
**Ethnicity, n (%)**						0.089
	Jewish	10,577 (84.4)	7465 (87.2)	9225 (82.1)		
	Arab-Bedouin	1958 (15.6)	1096 (12.8)	1966 (17.5)		
**Immigration status, n (%)**						0.195
	<1991	4300 (34.3)	3293 (38.5)	3034 (27)		
	>1991	2588 (20.6)	1360 (15.9)	2005 (17.8)		
	Nonimmigrants	5648 (45.1)	3908 (45.6)	6202 (55.2)		
**Educational status, n (%)**						0.049
	Elementary	1201 (9.6)	875 (10.2)	987 (8.8)		
	High school	3338 (26.6)	2513 (29.4)	2485 (22.1)		
	University	2614 (20.9)	1917 (22.4)	1778 (15.8)		
**Marital status, n (%)**						0.068
	Single	823 (6.6)	596 (7)	849 (7.6)		
	Married	8014 (63.9)	5571 (65.1)	6574 (58.5)		
	Divorced	1055 (8.4)	643 (7.5)	760 (6.8)		
	Widowed	1056 (8.4)	852 (10)	904 (8)		
**Age-adjusted Charlson Comorbidity Index**						0.328
	Mean (SD)	4.33 (3.09)	4.66 (3.19)	3.21 (2.70)		
	Median (IQR)	4.00 (2-6)	4 (2-6)	2 (1-4)		
Smoking, n (%)		3884 (31)	2962 (34.6)	3436 (30.6)	0.026	
COVID-19 infection, n (%)		758 (6)	618 (7.2)	824 (7.3)	0.034	
Number of COVID-19 of vaccinations, median (IQR)		3 (0-3)	3 (2-3)	2 (0-3)	0.209	
**Annual number of primary care physician visits, median (IQR)**						
	2019	15 (10-21)	16 (11-23)	13 (8-19)	0.233	
	2020-2021	14 (9.5-20)	16 (11-23)	11 (6.5-16.5)	0.379	

^a^SMD: standardized mean difference.

**Figure 3:**
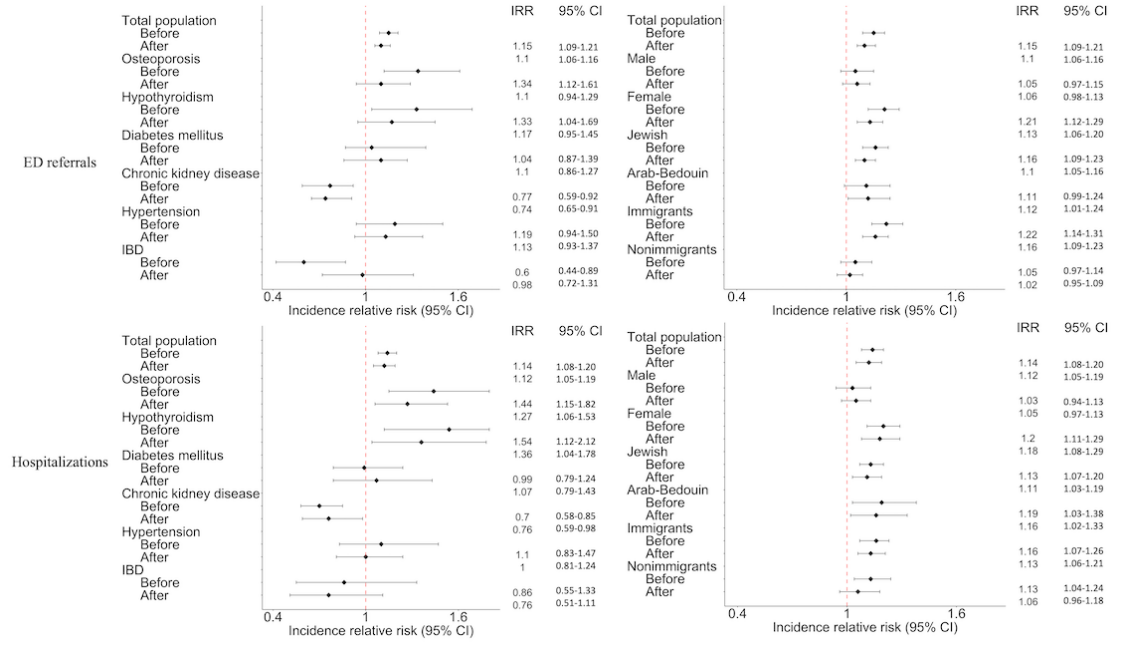
Multivariable regression models—an independent association of telemedicine versus physical in-person patients; the analysis is stratified by comorbidity and demographic characteristics, before and after the telemedicine surge. ED: emergency department; IBD: inflammatory bowel disease; IRR: incidence rate ratio.

Figure 3 shows the results of a multivariable regression model analysis comparing telemedicine and in-person patients, adjusted for demographic characteristics, physician characteristics, medical field, chronic disease monitoring measurements, and COVID-19–related behaviors (study outcomes without adjustment are shown in Multimedia Appendix 1). In 2019, before the telemedicine surge, patients in the total population had a higher IRR in ED referrals and hospitalization (IRR 1.15, 95% CI 1.09-1.21 and IRR 1.14, 95% CI 1.08-1.20, respectively). During 2020-2021, after the telemedicine surge, the IRR remained stable, indicating that telemedicine use was not associated with an increased risk of ED referrals or hospitalizations compared with in-person visits (IRR 1.1, 95% CI 1.06-1.16 and IRR 1.12, 95% CI 1.05-1.19, respectively).

In the subgroup analysis by comorbidities and demographic characteristics, it was found that in 2019, telemedicine patients with osteoporosis had a higher IRR in hospitalizations than in-person patients (IRR 1.44, 95% CI 1.15-1.82). This IRR decreased during 2020-2021 (IRR 1.27, 95% CI 1.06-1.53). Similarly, the IRR in ED referrals in 2019 (IRR 1.34, 95% CI 1.12-1.61) decreased during 2020-2021 (IRR 1.1, 95% CI 0.94-1.29), narrowing the gap between the groups. A similar pattern was observed in patients with hypothyroidism showing a relative decrease in IRR for hospitalizations (IRR 1.36, 95% CI 1.04-1.78) and ED referrals (IRR 1.17, 95% CI 0.95-1.45) during 2020-2021 compared with 2019 (IRR 1.54, 95% CI 1.12-2.12 and IRR 1.33, 95% CI 1.04-1.69, respectively). This trend was also observed in the IRR for hospitalization among patients with inflammatory bowel disease and hypertension, showing a narrowing of the gap between the groups during 2020-2021 (IRR, 0.76, 95% CI 0.51-1.11 and IRR 1.0, 95% CI 0.81-1.24, respectively) compared with 2019 (IRR 0.86, 95% CI 0.55-1.33 and IRR 1.1, 95% CI 0.83-1.47, respectively).

Tables S1-2 in Multimedia Appendix 2 present a subgroup analysis stratified by comorbidity among two main specialties—endocrinology and nephrology—focusing on four chronic conditions: hypothyroidism, diabetes mellitus, chronic kidney disease, and hypertension, along with their appropriate measurements for evaluation. These parameters were analyzed and compared between 2019 and 2020-2021, before the telemedicine surge and after, across three groups: in-person visits; remote consultations; and no visits, which is the control group. The control group included patients who did not attend any medical consultations during the pandemic, representing the typical course of chronic conditions without medical assistance. In this analysis, both in-person and telemedicine patients with hypothyroidism showed a decrease in levels of TSH from 2019 to 2020-2021 (from 4.36 to 4.09 and from 5.06 to 5.04, respectively) and an increase in levels of T4 (from 1.24 to 1.29 and from 1.26 to 1.31, respectively). Regarding patients with diabetes mellitus, both in-person and telemedicine patients demonstrated a comparable decrease in HbA_1c_ levels during 2020-2021 (from 7.81 to 7.64 and from 8.41 to 7.85, respectively). Notably, patients with chronic kidney disease in all groups had an increase in creatinine levels during 2020-2021, with a slightly larger increment in the in-person patient group.

The characteristics of physicians providing physical, in-person care versus telemedicine consultations are summarized in Table 2. Telemedicine physicians were older, with a mean age of 50.6 (SD 9.29) years compared with 40.3 (8.21) years for in-person physicians (SMD=0.668). A higher proportion of telemedicine physicians were specialists (69/80, 86.3% vs 14/21, 66.7%; SMD=0.474). Regarding the field of medicine, telemedicine physicians were more frequently involved in hemato-oncology (37/80, 46.2% vs 3/21, 14.3%), while in-person physicians were more often from gastroenterology (11/21, 52.4% vs 16/80, 20%). Other fields, such as endocrinology, nephrology, and psychiatry, showed a more balanced distribution, although differences were noted.

**Table 2:** Physician characteristics.

Characteristics			Physical, in-person physicians (n=21)	Telemedicine physicians (n=80)	SMD^a^	
**Age (years)**						0.668
		Mean (SD)	40.3 (8.21)	50.6 (9.29)		
		Median (IQR)	40 (32-51)	50 (38-65)		
**Sex, n (%)**						0.190
	Female		7 (33.3)	34 (42.5)		
**Professional career status, n (%)**						0.474
		Specialist	14 (66.7)	69 (86.3)		
		Intern	7 (33.3)	11 (13.8)		
**Distance from hospital, n (%)**						0.745
		Hospital town residents	4 (19)	42 (52.5)		
		Non–hospital town residents	17 (81)	38 (47.5)		
**Field of medicine, n (%)**						1.110
		Hemato-oncology	3 (14.3)	37 (46.2)		
		Gastro	11 (52.4)	16 (20)		
		Endocrinology	1 (4.8)	9 (11.3)		
		Nephrology	1 (4.8)	9 (11.3)		
		Psychiatry	5 (23.8)	9 (11.3)		

^a^SMD: standardized mean difference.

Multimedia Appendix 3 stratifies physicians based on their telemedicine use in 2019 and during 2020-2021. Physicians were categorized into 4 groups based on the percentage increase in their telemedicine usage during 2020-2021: Q1 (<10% use), Q2 (10%-20% use), Q3 (20%-33% use), and Q4 (>33% use). Notably, as telemedicine use increased across quartiles, the average number of ED referrals and hospitalizations per patient decreased. For ED referrals, the mean values were 0.53 (SD 1.49) for Q1, 0.47 (SD 1.26) for Q2, 0.40 (SD 1.14) for Q3, and 0.36 (SD 1.38) for Q4. For hospitalizations, the mean values were 0.31 (SD 0.96) for Q1, 0.26 (SD 1.01) for Q2, 0.15 (SD 0.76) for Q3, and 0.15 (SD 0.78) for Q4. Multivariable regression models were used to further analyze the data, and a similar impact was observed, with lower IRR for ED referrals and hospitalization in the Q4 group of physicians, that is, those with the highest use percentage (IRR 0.85, 95% CI 0.79-0.91 and IRR 0.78 95% CI 0.72-0.84, respectively; *P*<.001).

Multimedia Appendix 4 presents multivariable regression models comparing telemedicine and physical, in-person consultations for ED referrals and hospitalizations, stratified by physicians’ characteristics in 2019 and during 2020-2021. Telemedicine was associated with a reduction in ED referrals during 2020-2021 in the total population (IRR 0.57, 95% CI 0.37-0.90), particularly among female physicians (IRR 0.38, 95% CI 0.19-0.74). Additionally, a notable reduction in hospitalizations was observed among patients under the care of telemedicine physicians during 2020-2021 in the total population (IRR 0.7, 95% CI 0.40-1.28).

## Discussion

### Principal Findings

This study aimed to evaluate the impact of telemedicine on health care delivery and patient outcomes. The COVID-19 pandemic has led to unprecedented changes in health care delivery, particularly the promotion and accelerated implementation of telemedicine [8]. Our analyses revealed that in 2019, only 0.4% (332/83,000) of patients used telemedicine, while in 2020, this proportion increased to 22.1% (14,839/67,213) of patients opting for telemedicine visits across various medical specialties. This dramatic shift, driven by the pandemic’s conditions and constraints, can be viewed as a “natural experiment” where the population transitioned to remote care with minimal selection bias.

Our findings suggest that telemedicine is a viable method for delivering outpatient care across different fields of medicine, potentially comparable to in-person care. However, careful consideration is needed based on each patient’s disease and characteristics. Our data showed that telemedicine patients were slightly older and had a higher comorbidity burden than those who used in-person visits. While we initially expected that younger patients would be more agile and easily adopt technology for medical care [16,17], we found that telemedicine patients were in fact older. They may reflect mobility issues or transportation challenges that motivated older people to seek care from home. Additionally, male patients used telemedicine visits more frequently than female patients. Recent studies have researched how gender may impact the use of telemedicine, yet findings have been inconsistent. Hargittai and Shafer [18] found that women’s lower self-assessment regarding their digital skills affects the extent of use, which could possibly explain our results.

Patients of Jewish ethnicity showed a preference for telemedicine visits compared with Arab-Bedouin ethnicity. This finding may be attributed to language barriers, communication difficulties with health care providers, or disparities in internet and technology access between Jewish– and Arab-Bedouin–based communities in southern Israel. This aligns with previous studies of factors associated with using telemedicine in Israel during the pandemic. For example, one study on the implementation of telemedicine showed that ethnicity was the most significant factor associated with telemedicine use, with telemedicine use being 85% and 52% among Jewish and Arab-Bedouin patients, respectively [19]. Despite these variations, telemedicine emerged as a feasible alternative for delivering care, demonstrating its potential for managing outpatients with chronic disease.

The analysis of chronic disease measurements across various specialties provided insights into the role of telemedicine in the management of different diseases. Patients with hypothyroidism demonstrated comparable changes in TSH and T4 levels after the increase in telemedicine use during 2020-2021, regardless of whether they received in-person or telemedicine consultations. This finding aligns with existing literature, indicating that telemedicine facilitates timely monitoring of thyroid function and hormone adjustments, which enhances adherence and minimizes delays in care [20]. Similarly, patients with diabetes mellitus exhibited a decrease in HbA_1c_ levels during 2020-2021, with no statistically significant difference between in-person and telemedicine patients. Remote consultations have been shown to optimize glycemic control by reducing HbA_1c_ levels and improving medication adherence, particularly for patients with poorly controlled diabetes. The American Diabetes Association highlights that telehealth enhances glycemic management in patients with unmanaged diabetes by providing continuous support and facilitating frequent interactions [21,22]. These findings suggest that telemedicine can support disease management for certain chronic conditions, consistent with other recent studies [7,16]. A recent large-scale umbrella review of clinical outcomes of telemedicine interventions for various conditions, including diabetes management, with a focus on glycemic control assessed by HbA_1c_ measurements, concluded that telemedicine was comparable or superior to in-person interventions [6]. Furthermore, in our study, we assessed kidney function by measurements of creatinine levels. During 2020-2021, creatinine levels increased in both telemedicine and in-person patients, with a slightly larger increment observed in the latter group. This finding suggests the noninferiority of remote care compared with in-person care of renal function. These results align with previous studies that demonstrated specialist care for patients with renal disease via telemedicine was feasible and sustainable with comparable outcomes to those of in-person consultations [23,24]. “Telenephrology” has been shown to improve disease management by enabling early detection of complications, optimizing medication regimens, and providing dietary and lifestyle counseling. The Kidney Disease: Improving Global Outcomes guidelines endorse telehealth to expand access and improve chronic kidney disease care [25,26].

The multivariable regression model analysis of ED referrals and hospitalizations before and after the telemedicine surge, adjusted for demographic characteristics, physician characteristics, field of medicine, chronic disease monitoring measurements, and COVID-19 protective behaviors, showed no evidence of worse outcomes for telemedicine users compared with in-person visits (Figure 3). In 2019, before the surge in telemedicine use, telemedicine patients had a higher IRR for hospitalizations and ED referrals. During 2020-2021, these IRRs remained stable, indicating that telemedicine was not associated with an increased risk of hospitalizations or ED referrals compared with in-person care. This finding aligns with previous research showing that telemedicine was associated with a reduction in physical, in-person consultations while maintaining care quality during the COVID-19 pandemic [6,27]. We assessed the possibility that our results were confounded by COVID-19–related behaviors, meaning that telemedicine patients were less keen to visit health care facilities due to the risk of infection during the pandemic. However, our analysis found no substantial differences between the groups in the number of vaccinations and primary care physician visits before and during the pandemic. Furthermore, our analysis models, which were adjusted to these parameters, minimized the possibility that patient behavior during COVID-19 influenced our observations. By incorporating these adjustments, we ensured that the results are comparable despite varying social and health environments of the prepandemic and pandemic periods.

Notably, patients with osteoporosis, hypothyroidism, inflammatory bowel disease, and hypertension did not experience an increased risk of hospitalization or ED referrals during 2020-2021 compared with 2019. These findings suggest that telemedicine may serve as a viable alternative to in-person care for managing these chronic conditions without evidence of worsening outcomes.

The analysis of demographic characteristics revealed more insights regarding the impact of telemedicine in specific populations. For example, nonimmigrants who opt for telemedicine visits had a lower RR of ED referrals and hospitalization. Presumably, this could be explained by potential communication barriers between patients and health care providers among immigrants due to language or cultural differences [18].

This study also explored the impact of health care providers’ perspectives. While most studies of telemedicine focus on patient perspectives and satisfaction, provider experience has been less studied. Previous studies investigating the provider perspective found that provider satisfaction is a critical determinant of whether telemedicine will be used to see patients [28]. Moreover, it was found that for telemedicine to be successful, the technology must be reliable and providers need adequate training [29]. Another study emphasized that telemedicine’s success hinges on the willingness of both providers and patients to use it. Provider acceptance emerged as the most important factor determining success. In fact, high acceptance could overcome other obstacles such as low demand, technology problems, and insufficient financing [30]. Our analysis revealed an association between increased telemedicine utilization and decreased rates of ED referrals and hospitalizations (Tables S1-2 in Multimedia Appendix 2). In Table 2, the analysis of the physician characteristics showed telemedicine physicians were older and more likely to be specialists. This might reflect a preference for telehealth among more experienced physicians, possibly due to greater comfort with managing complex cases remotely. These findings support the notion that telemedicine can sustain quality care delivery without increasing acute care visits, even for patients with comorbidities.

While our study primarily focused on the short-term impacts of telemedicine during the pandemic, we acknowledge the importance of long-term follow-up to evaluate its sustained impact and potential challenges. Long-term studies could provide valuable insights into whether the initial benefits of telemedicine, such as reduced acute care visits and improved chronic disease management, are maintained over time. Additionally, such research could identify potential drawbacks, such as technology fatigue, disparities in access, or long-term patient satisfaction trends. Future research should explore the integration of telemedicine with other care modalities, its impact on health outcomes beyond acute care visits, and its role in addressing evolving health care challenges.

This study has several limitations. First, it does not include data for private medical services, which are a viable option for receiving medical care, as appointments may be conducted in person or remotely. Missing private medical data from the analysis could potentially lead to an underestimation of telemedicine use in our cohort, as private health care providers may have different adoption rates and practices for remote care. However, the proportion of private health care use in the southern region of Israel is small, and its influence is most probably negligible. Second, in addition to the medical threats posed by the COVID-19 pandemic, it brought about other challenges affecting various aspects of the population’s mental and physical well-being. Therefore, the transition to telemedicine may implicitly include other important factors such as loneliness, depression, physical deterioration, changes in the presence of children in the household, and more. Additionally, the retrospective design and reliance on electronic medical records could introduce potential biases and limitations in data collection and analysis such as incomplete data capture or misclassification. These factors could affect the accuracy of our estimates of telemedicine’s impact on clinical outcomes. Despite these limitations, we believe the large sample size and comprehensive nature of our dataset provide valuable insights into telemedicine’s role in chronic disease management.

### Conclusions

Our findings support the potential role of telemedicine in achieving comparable clinical outcomes to in-person visits for managing various medical conditions. The results of this research highlight the potential of telemedicine to enhance health care delivery without compromising medical outcomes, particularly for specific populations and conditions. Although the timing of infectious pandemics is unpredictable, their recurrence is probable. It is evident that telemedicine has a critical role in emergency responses, underscoring its importance. Our findings emphasize the importance of integrating telemedicine into health care systems and policies to ensure consistent patient outcomes across various situations and optimize health care resource allocation. This study also highlights the importance of considering individual patient suitability when considering telemedicine care. Further research is necessary to optimize the implementation and potential role of telemedicine in achieving comparable clinical outcomes, tailoring it to the unique characteristics and needs of each patient.

## Appendix

Multimedia Appendix 1 Study outcomes without adjustment.

Multimedia Appendix 2 Comorbidity stratification—analysis of endocrinology outcomes.

Multimedia Appendix 3 Medical physicians stratification by telemedicine usage during 2020-2021.

Multimedia Appendix 4 Multivariable regression models—an independent association of telemedicine versus physical, in-person patients; the analysis is stratified by physicians’ characteristics, before and after the telemedicine surge. The adjustment includes physician characteristics (age, sex, carreer status, and distance from hospital) and field of medicine. ED: emergency department; IRR: incidence rate ratio.

## Abbreviations

CCI: Charlson Comorbidity Index
DAG: directed acyclic graph
ED: emergency department
HbA1c: hemoglobin A1c
IRR: incidence rate ratio
SMD: standardized mean difference
SUMC: Soroka University Medical Center
T4: thyroxine
TSH: thyroid-stimulating hormone

## Data Availability

The datasets generated or analyzed during this study are available from the corresponding author on reasonable request.
