# Supplementary material for: Evaluating Clinical Outcomes and Physician Adoption of Telemedicine for Chronic Disease Management: Population-Based Retrospective Cohort Study
Source: J Med Internet Res. 2025 Apr 28;27:e66499. doi: 10.2196/66499 (PMC12070016; doi:10.2196/66499)
Supplement: Multimedia Appendix 1 [file jmir_v27i1e66499_app1.docx]

**Multimedia Appendix 1.** Study outcomes without adjustment.

|  | Physical In-Person Patients (N=12,536) | Telemedicine Patients (N=8,561) | Control Group (N=11,348) | SMD |
| --- | --- | --- | --- | --- |
| **ED Referrals 2019 -** Mean (SD) | 0.733 (1.57) | 0.834 (2.19) | 0.798 (1.95) | 0.036 |
| **ED Referrals 2020 -** Mean (SD) | 0.604 (1.22) | 0.707 (1.45) | 0.528 (1.07) | 0.094 |
| **Hospitalizations 2019 -** Mean (SD) | 0.483 (1.21) | 0.530 (1.24) | 0.428 (1.11) | 0.057 |
| **Hospitalizations 2020 -** Mean (SD) | 0.378 (0.806) | 0.454 (0.937) | 0.252 (0.604) | 0.174 |
