# Supplementary material for: Evaluating Clinical Outcomes and Physician Adoption of Telemedicine for Chronic Disease Management: Population-Based Retrospective Cohort Study
Source: J Med Internet Res. 2025 Apr 28;27:e66499. doi: 10.2196/66499 (PMC12070016; doi:10.2196/66499)
Supplement: Multimedia Appendix 2 [file jmir_v27i1e66499_app2.docx]

**Multimedia Appendix 2.** Comorbidity stratification—analysis of endocrinology outcomes.

**Table S1.** Comorbidity Stratification –analysis of endocrinology outcomes

|  | | Endocrinology | | | | | | |
| --- | --- | --- | --- | --- | --- | --- | --- | --- |
|  |  | Hypothyroidism | | | Diabetes Mellitus | | | |
|  |  | Control Group | Physical In-Person Patients | Telemedicine Patients | Control Group | Physical In-Person Patients | Telemedicine Patients |  |
|  |  | **N=277** | **N=463** | **N=466** | **N=212** | **N=385** | **N=641** |  |
| **Age -** Mean (SD) | | 45.8 (20.0) | 58.1 (16.9) | 58.4 (17.4) | 58.2 (16.3) | 60.2 (14.7) | 60.4 (15.2) |  |
| **Gender –** Female, % | | 237 (85.6%) | 389 (84.0%) | 393 (84.3%) | 91 (42.9%) | 170 (44.2%) | 298 (46.5%) |  |
| **Ethnicity -** Jew , % | | 195 (70.4%) | 375 (81.0%) | 405 (86.9%) | 182 (85.8%) | 357 (92.7%) | 604 (94.2%) |  |
| **TSH, uU/mL-** Mean (SD) | Before Covid-19 | 7.41 (14.7) | 4.36 (7.59) | 5.06 (12.2) | **-** | **-** | **-** |  |
|  | During Covid-19 | 5.56 (9.11) | 4.09 (6.90) | 5.04 (11.1) |  |  |  |  |
| **T3, ng/dL-** Mean (SD) | Before Covid-19 | 2.84 (0.551) | 2.94 (0.672) | 2.93 (1.07) | **-** | **-** | **-** |  |
|  | During Covid-19 | 3.01 (0.616) | 3.01 (0.501) | 2.96 (0.474) |  |  |  |  |
| **T4, μg/dL-** Mean (SD) | Before Covid-19 | 1.15 (0.250) | 1.24 (0.234) | 1.26 (0.429) | **-** | **-** | **-** |  |
|  | During Covid-19 | 1.19 (0.240) | 1.29 (0.252) | 1.31 (0.266) |  |  |  |  |
| **HA1C, %-** Mean (SD) | Before Covid-19 | **-** | **-** | **-** | 7.82 (1.71) | 7.81 (1.53) | 8.41 (11.9) |  |
|  | During Covid-19 |  |  |  | 7.87 (1.61) | 7.64 (1.37) | 7.85 (2.16) |  |
|  | During Covid-19 |  |  |  |  |  |  |  |

**Table S2.** Comorbidity Stratification –analysis of nephrology outcomes

|  | | Nephrology | | | | | |
| --- | --- | --- | --- | --- | --- | --- | --- |
|  |  | Chronic Kidney Disease | | | Hypertension | | |
|  |  | Control Group | Physical In-Person Patients | Telemedicine Patients | Control Group | Physical In-Person Patients | Telemedicine Patients |
|  |  | **N=193** | **N=379** | **N=601** | **N=222** | **N=382** | **N=464** |
| **Age -** Mean (SD) | | 68.7 (16.1) | 66.4 (14.7) | 69.0 (13.9) | 66.0 (15.4) | 67.5 (12.8) | 69.1 (12.7) |
| **Gender –** Female, % | | 76 (39.4%) | 152 (40.1%) | 231 (38.4%) | 123 (55.4%) | 196 (51.3%) | 238 (51.3%) |
| **Ethnicity -** Jew , % | | 150 (77.7%) | 271 (71.5%) | 491 (81.7%) | 181 (81.5%) | 301 (78.8%) | 374 (80.6%) |
| **Creatinine, mg/dL-** Mean (SD) | Before Covid-19 | 2.15 (1.43) | 2.59 (1.95) | 1.96 (1.10) | **-** | **-** | **-** |
|  | During Covid-19 | 2.57 (2.15) | 3.21 (2.50) | 2.30 (1.49) |  |  |  |
| **Urine Microalbumin/ Creatinine, mg/g-** Mean (SD) | Before Covid-19 | 677 (1440) | 704 (967) | 632 (804) | **-** | **-** | **-** |
|  | During Covid-19 | 415 (887) | 688 (1120) | 640 (928) |  |  |  |
| **Systolic BP, mmHg-** Mean (SD) | Before Covid-19 | **-** | **-** | **-** | 142 (15.7) | 144 (15.7) | 142 (16.1) |
|  | During Covid-19 |  |  |  | 139 (19.2) | 138 (12.6) | 140 (22.8) |
| **Diastolic BP, mmHg -** Mean (SD) | Before Covid-19 | **-** | **-** | **-** | 75.3 (12.2) | 73.8 (12.5) | 76.0 (12.4) |
|  | During Covid-19 |  |  |  | 71.5 (14.4) | 68.4 (11.9) | 73.5 (15.7) |
