# Supplementary material for: Evaluating Clinical Outcomes and Physician Adoption of Telemedicine for Chronic Disease Management: Population-Based Retrospective Cohort Study
Source: J Med Internet Res. 2025 Apr 28;27:e66499. doi: 10.2196/66499 (PMC12070016; doi:10.2196/66499)
Supplement: Multimedia Appendix 3 [file jmir_v27i1e66499_app3.docx]

**Multimedia Appendix 3.** Medical physicians stratification by telemedicine usage during 2020-2021.

| **Telemedicine Usage Increase During COVID-19** | | | | | |
| --- | --- | --- | --- | --- | --- |
|  | **Q1 Physicians <10%** | **Q2 Physicians 10-20%** | **Q3 Physicians 20-33%** | **Q4 - Physicians >33%** | **SMD** |
|  | **N= 37 Physicians 4394 patients** | **N=37 Physicians 3979 patients** | **N=37 Physicians 5715 patients** | **N=37 Physicians 4208 patients** |  |
| **ED Referrals per patient during 2020-2021** | | | | | |
| Mean (SD) | 0.53 (1.49) | 0.47 (1.26) | 0.40 (1.14) | 0.36 (1.38) | 0.071 |
| Median [Q1, Q3] | 0.50 [0, 1.0] | 0 [0, 1.0] | 0 [0, 1.0] | 0 [0, 1.0] |  |
| **Hospitalizations per patient during 2020-2021** | | | | | |
| Mean (SD) | 0.31 (0.96) | 0.26 (1.01) | 0.15 (0.76) | 0.15 (0.78) | 0.112 |
| Median [Q1, Q3] | 0 [0, 0.50] | 0 [0, 0.50] | 0 [0, 0.50] | 0 [0, 0.50] |  |
| **Multi-variable regression models; stratified analysis by Medical Physicians Telemedicine Usage During 2020-2021.** | | | | | |
|  |  |  | **IRR***^1^* | **95% CI***^1^* | ***P*** |
| **ED Referrals** | Q1 Physicians <10% | | — | — |  |
|  | Q2 Physicians 10-20% | | 0.94 | 0.88, 1.00 | 0.055 |
|  | Q3 Physicians 20-33% | | 0.90 | 0.85, 0.96 | 0.001 |
|  | Q4 - Physicians >33% | | 0.85 | 0.79, 0.91 | <0.001 |
| **Hospitalizations** | Q1 Physicians <10% | | — | — |  |
|  | Q2 Physicians 10-20% | | 0.95 | 0.88, 1.02 | 0.140 |
|  | Q3 Physicians 20-33% | | 0.79 | 0.73, 0.84 | <0.001 |
|  | Q4 - Physicians >33% | | 0.78 | 0.72, 0.84 | <0.001 |

IRR: incidence rate ratio.

The adjustment includes age, sex, ethnicity, and Charlson Comorbidity Index.
