# Supplementary figures and images for: Evaluating Clinical Outcomes and Physician Adoption of Telemedicine for Chronic Disease Management: Population-Based Retrospective Cohort Study
Source: J Med Internet Res. 2025 Apr 28;27:e66499. doi: 10.2196/66499 (PMC12070016; doi:10.2196/66499)

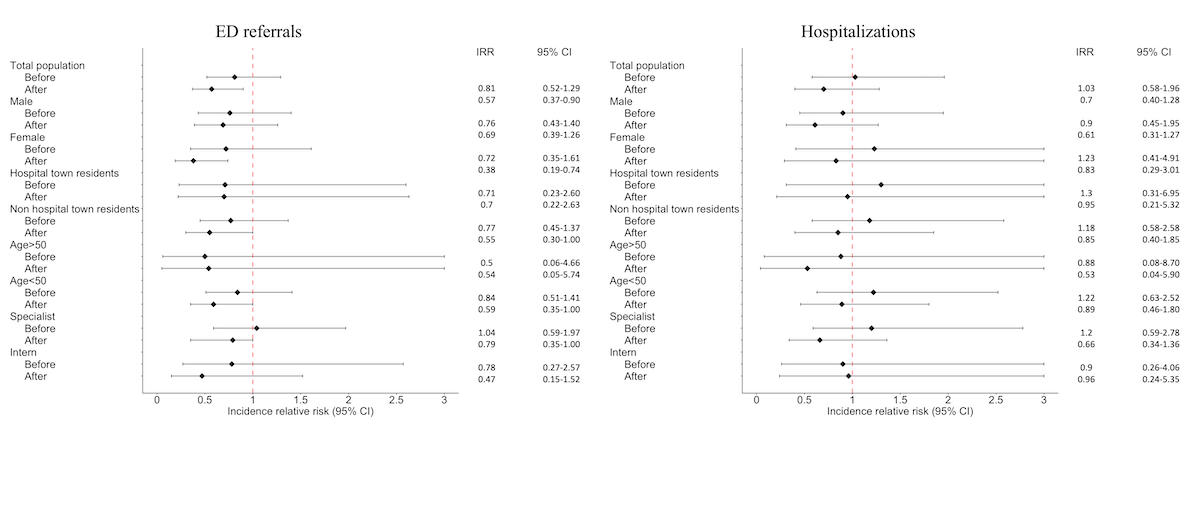

Supplement: Multimedia Appendix 4 [file jmir_v27i1e66499_app4.png]
